# Influence of Storage Time and Temperature on the Toxicity, Endocrine Potential, and Migration of Epoxy Resin Precursors in Extracts of Food Packaging Materials

**DOI:** 10.3390/molecules24234396

**Published:** 2019-12-02

**Authors:** Błażej Kudłak, Natalia Jatkowska, Paweł Kubica, Galina Yotova, Stefan Tsakovski

**Affiliations:** 1 Department of Analytical Chemistry, Faculty of Chemistry, Gdańsk University of Technology, 11/12 Naturowicza, 80-233 Gdańsk, Poland; blakudla@pg.edu.pl (B.K.); pawkubic@pg.edu.pl (P.K.); 2 Chair of Analytical Chemistry, Faculty of Chemistry and Pharmacy, Sofia University “St. Kliment Ohridski”, Sofia 1164, Bulgaria; galina_yotova@abv.bg (G.Y.); stsakovski@chem.uni-sofia.bg (S.T.)

**Keywords:** packaging, modeling, extraction, ecotoxicity, instrumental studies, analysis of variance simultaneous component analysis

## Abstract

The aim of the present study was to establish a standard methodology for the extraction of epoxy resin precursors from several types of food packages (cans, multi-layered composite material, and cups) with selected simulation media (distilled water, 5% ethanol, 3% dimethyl sulfoxide, 5% acetic acid, artificial saliva) at different extraction times and temperatures (factors). Biological analyses were conducted to determine the acute toxicity levels of the extracts (with *Vibrio fischeri* bacteria) and their endocrine potential (with *Saccharomyces cerevisiae* yeasts). In parallel, liquid chromatography-tandem mass spectrometry was performed to determine levels of bisphenol A diglycidyl ether (BADGE), bisphenol F diglycidyl ether (mixture of isomers, BFDGE), ring novolac glycidyl ether (3-ring NOGE), and their derivatives. The variation induced by the different experimental factors was statistically evaluated with analysis of variance simultaneous component analysis (ASCA). Our findings demonstrate the value of using a holistic approach to best partition the effects contributing to the end points of these assessments, and offer further guidance for adopting such a methodology, thus being a broadly useful reference for understanding the phenomena related to the impacts of food packaging materials on quality for long- and short-term storage, while offering a general method for analysis.

## 1. Introduction

Advances in food storage technology have greatly contributed to an overall increase of social well-being and global development. Indeed, packaging has vastly evolved from simple liquid or loose/powdery material containers into very sophisticated and dedicated systems enabling the long-term storage of specific products and utensils. The development of packaging must consider the high stability and quality of any material that will make direct contact with food or is destined to contain food prepared for a long shelf life [1]. Early studies of the integrity of semi-rigid packages used *Enterobacter aerogenes* and spores of *Bacillus subtilis* to confirm the high utility of the material, demonstrating results comparable to more traditional electrolytic tests [2]. Pet et al. evaluated the possibility of food spoilage and contamination with bacteria due to handling operations [3]. Nevertheless, additional tests and steps are required before any material can be considered as safe with fully known specifications and to sufficiently ensure the high quality of the material for its purpose in food contact and storage [4]. This requirement led to the introduction of proper legislation such as Regulations (EC) no. 1935/2004, 2023/2006 on good manufacturing practices, 282/2008, 10/2011, Council Directive 84/500/EEC or Commission Regulation 450/2009 on active and intelligent packaging in the EU countries. However, instrumental research on the stability of packaging is still not sufficient on its own to assure the safety of a packaging material [5]. Moreover, currently used comparative analysis methods for any life cycle typically involve screening analyses of the material used for packaging production without taking into account its potential shelf-life degradation [6].

Hence, additional methodologies are required to increase the safety measures in the packaging industry, including introducing simulation media into extraction schemes or using toxicological tools at the evaluation stage with advanced chemometric data treatment for appropriate interpretation [5]. Such a comprehensive and multifaceted approach would have the greatest potential to holistically describe the migration of plausible non-intentionally added substances (NIAS) and their transformation products when studying the effects of the short- and long-term storage of food products. Numerous studies have revealed the presence of some NIAS in both food and packaging materials. However, very few of these studies utilized both instrumental and biological methods to assess the combined effect of the xenobiotics present. To overcome this limitation, there have been many attempts to develop advanced extraction methods for holistically extracting analytes of interest from diverse materials [5] and determine the concentration levels of substances of interest (e.g., bisphenol A diglycidyl ether (BADGE), bisphenol F diglycidyl ether (BFDGE), and their hydroxyl and chlorinated derivatives) and their potential biological impacts.

As new materials are continuously being developed and used in the food industry, it has become increasingly important to study the potential impacts of novel composites that were previously out of reach using mechanical chemical synthesis. For example, the ordering and compaction of cellulose and collagen microfibers in nanomaterials with organic polymers, such as those developed by [7], were found to be affected by the presence of nano silver, thus altering the bactericidal and fungicidal properties of both materials. In addition, Chamorro-Garcia et al. [8] confirmed the adipogenic capacity of BADGE and BPA, and evaluated their effects on adipogenesis, osteogenesis, gene expression, and nuclear receptor activation in multipotent mesenchymal stromal stem cells. Thus, combining different materials in composite packaging has become a global trend. Accordingly, analytical methods must be developed to provide reasonable and reliable data for the holistic assessment of the stability and safety of these materials for customer assurance.

In this regard, the aim of the present study was to adopt an up-to-the-date approach for studying the quality of packaging for food storage utilizing instrumental, biological, and chemometric tools. For quantitative analysis, we adopted the analysis of variance simultaneous component analysis (ASCA) modeling approach, which was used as a last step of complex research on extractions from packaging materials (cans, cups, and multi-layered composite materials) treated with selected simulation media (under varying external conditions) to separate the relative effects. We assessed the biological activity of extracts with respect to acute toxicity evaluations (using the Microtox assay) and endocrine disruption potential (with XenoScreen), and conducted instrumental analysis of the potential contaminants with liquid chromatography coupled to tandem mass spectrometry (LC-MS/MS). The main objectives of this study were to statistically determine if an elaborated and integrated methodology could enable the identification of specific storage/extraction parameters with significant influences on observable toxicity and endocrine disruption potential, along with determination of the analytes responsible.

## 2. Results

### 2.1. ASCA Modeling of Can Extracts

The results from ASCA models for can lining extracts with all simulants are presented in Table 1. The main factors (temperature and contact time) and their interactions were all statistically significant for all ASCA models (*p* < 0.01). However, the partitioning of total variance for all ASCA models, except for that with 5% DMSO, indicated the stronger influence of temperature and the interaction between time and temperature compared to the main effect of contact time. 

**Figure 1:**
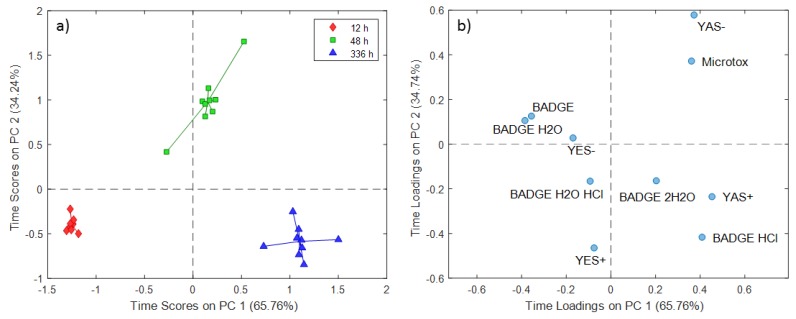
Simultaneous component analysis (SCA) time scores (**a**) and loadings (**b**) plot for distilled water extraction experiments.

The time scores and loading plots for distilled water extraction experiments are presented in Figure 1, demonstrating that the PC1 scores explained 65.76% of the total variance and the PC2 scores explained the remaining 34.24%. PC1 scores showed time-dependent variations in the response data from 12 h to 336 h, whereas the PC2 scores reflected specific effects observed only for experiments conducted at a contact time of 48 h, compared to 12 and 336 h (Figure 1a).

**Figure 2:**
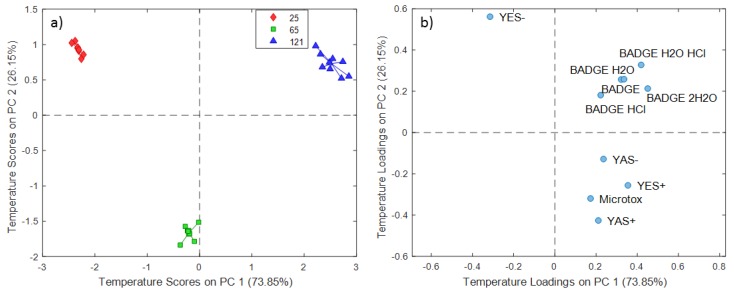
SCA temperature scores (**a**) and loadings (**b**) plot for distilled water extraction experiments.

The temperature scores and loadings plots for distilled water extraction experiments are presented in Figure 2, demonstrating that PC1 explained 73.85% of the total variance and PC2 explained the remaining 26.15%. Similar to the time scores plot, PC1 scores indicated temperature dependence from 25 °C to 121 °C, whereas the PC2 scores reflected the effect on extracts specifically obtained at 65 °C (Figure 2a).

**Figure 3:**
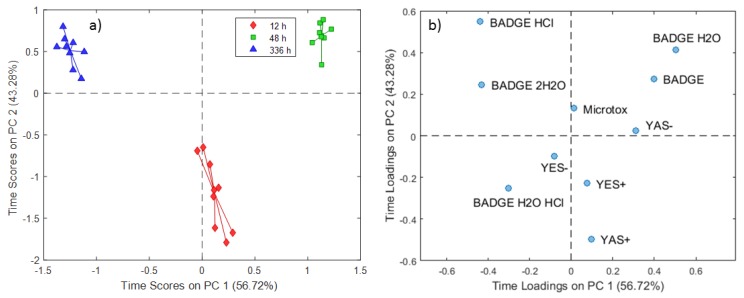
SCA time scores (**a**) and loadings (**b**) plot for 5% ethanol extraction experiments.

**Figure 4:**
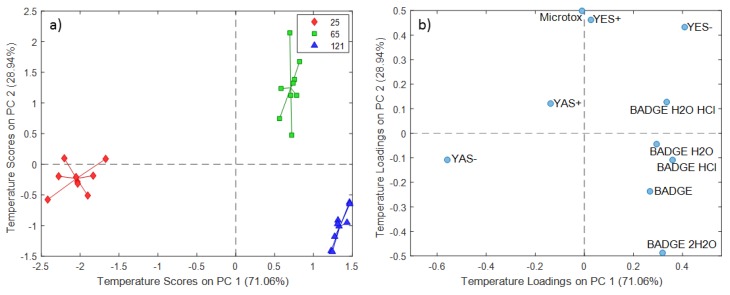
SCA temperature scores (**a**) and loadings (**b**) plot for 5% ethanol extraction experiments.

The ASCA model results for the 5% ethanol extraction experiments are presented in Figure 3 and Figure 4. For the time scores, PC1 (56.72% of the total variance) clearly separated extracts subjected to 48 h of contact from those exposed for 336 h, and PC2 (43.28% of the total variance) scores differentiated extracts obtained at a 12 h contact time from the others (Figure 3a).

The temperature scores and loadings plots for the 5% ethanol extraction experiments are presented in Figure 4. The PC1 (71.06% of the total variance) scores demonstrated a temperature dependence from 25 °C to 121 °C, whereas the PC2 (28.94% of the total variance) scores differentiated extracts obtained at 65 °C from those obtained at 25 and 121 °C (Figure 4a).

**Figure 5:**
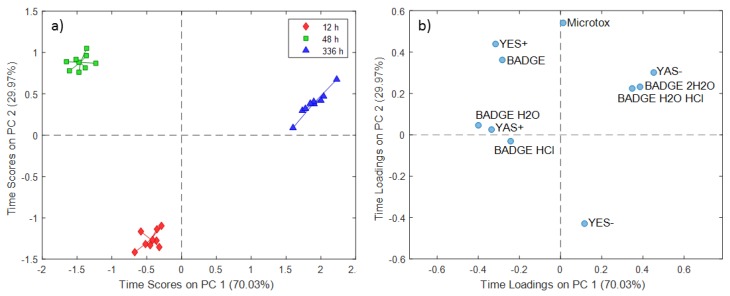
SCA time scores (**a**) and loadings (**b**) plot for 3% acetic acid extraction experiments.

**Figure 6:**
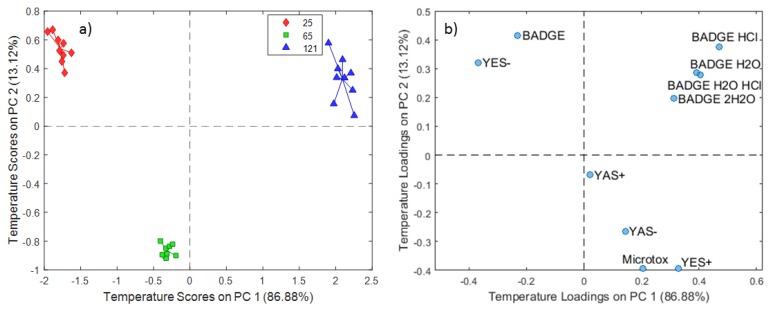
SCA temperature scores (**a**) and loadings (**b**) plot for 3% acetic acid extraction experiments.

In Figure 5 and Figure 6 the PC time and temperature scores plot for the 3% acetic acid extraction experiments are presented. The PC time scores and loadings plot outline some interesting associations between extracts obtained at different contact time and groups of similar variables (Figure 5) while the temperature plots (Figure 6) resemble the relation between migration of BADGE derivatives and temperature.

**Figure 7:**
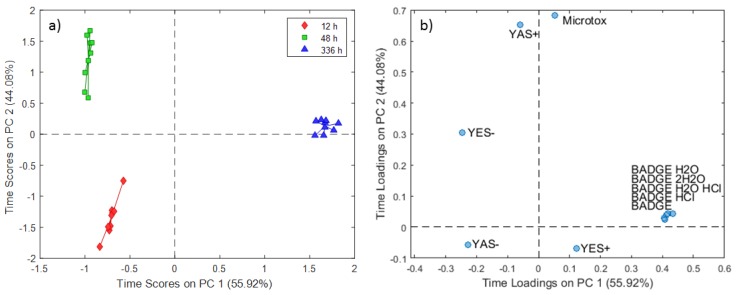
SCA time scores (**a**) and loadings (**b**) plot for 5% DMSO extraction experiments.

**Figure 8:**
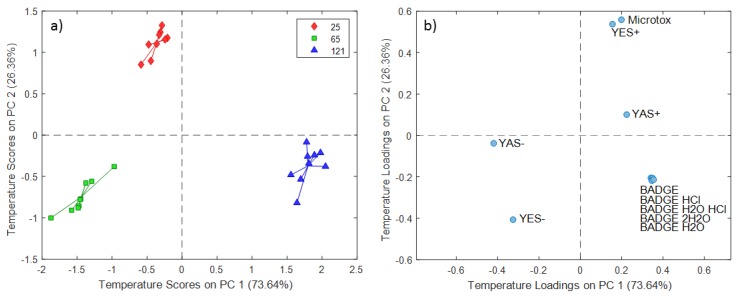
SCA temperature scores (**a**) and loadings (**b**) plot for 5% DMSO extraction experiments.

In Figure 7 and Figure 8 are PC scores obtained for 5% DMSO extracts. The obtained ASCA results outline the increased migration of BADGE and related compounds with the increase of contact time and temperature.

### 2.2. ASCA Modeling of Multi-Layered Composite Material Extracts

The results from ASCA models for multi-layered composite lining extracts with all simulants are presented in Table 2. The PC scores and loadings plots for all ASCA models are presented in Supplementary Figures S3–S10.

### 2.3. ASCA Modeling of Cup Extracts

The results from ASCA models for extracts of the external surface of cups obtained with distilled water, 5% ethanol, and 3% acetic acid are presented in Table 3. The PC scores and loadings plots for all ASCA models are presented in Supplementary Figures S11–S16.

## 3. Discussion

### 3.1. Discussion of ASCA Modeling of Can Extracts

A comparison between time scores and the loadings plot for distilled water extraction experiments (Figure 1b) indicates the relation between variables or a group of variables and experiments conducted at different contact times. The obtained scores and loadings demonstrated higher androgenic antagonistic activity (YAS-) and acute toxicity (Microtox) for experiments conducted at 48 h that could not be attributed to the investigated BADGE and its hydroxyl and chlorinated derivatives. The extracts of experiments conducted at prolonged contact time 336 h are characterized by androgenic agonistic activity (YAS+) which could be related to increased BADGE·HCl and BADGE·2H_2_O levels. Considering both the scores and loading plots (Figure 2b), there is an apparent relation of compounds from the BADGE group (BADGE and chlorinated and hydroxyl derivatives) with extracts obtained at 121 °C. These results suggested that the migration of BADGE and its derivatives compounds increases with increasing temperature.

Similar to the distilled water extraction experiments, the migration of BADGE·HCl and BADGE·2H_2_O (Figure 3b) increased at prolonged contact times, i.e., experiments conducted at 336 h for 5% ethanol extracts. Further, there was a relation between levels of BADGE or BADGE·H_2_O and experiments conducted at 48 h, indicating that migration of these BADGE and related compounds occurs at this contact time. However, the estrogenic and androgenic agonistic activity of extracts obtained at 12 h could not be attributed to the migration of this compounds. PC1 temperature loadings showed a strong positive relation with a YES- result and a strong negative relation with a YAS- result (Figure 4b). The positive PC1 temperature loadings further revealed that migration of these compounds increases with increasing temperature.

The PC time scores plot for the 3% acetic acid extraction experiments showed a clear distinction between experiments conducted at different contact times (Figure 5a). PC1 (70.03% of the total variance) differentiated extracts obtained at 48 h and 336 h, while PC2 (29.97%) distinguished the experiments conducted at 12 h from the others. The PC time loadings plot revealed some groups of variables related to endocrine disruption outputs and BADGE and its derivatives (Figure 5b). The group consisting of YES+ and BADGE could be related to the extracts obtained at a contact time of 48 h, since the group formed by YAS-, BADGE·2H_2_O, and BADGE·H_2_O·HCl could be related to experiments conducted at a prolonged contact time (336 h). The temperature score plot for the 3% acetic acid extraction experiments (Figure 6.) showed temperature dependence from 25 °C to 121 °C on PC1 (86.88% of the total variance), whereas the PC2 (13.12% of the total variance) scores differentiated extracts obtained at 65 °C specifically, compared to the other two treatments. The temperature loading plot confirmed that migration of BADGE and chlorinated and hydroxyl derivatives increases with the increase of temperature, excluding BADGE itself.

The ASCA model results for the 5% DMSO extraction experiments demonstrated a strong relation between the migration of analytes from BADGE group and contact time (Figure 7). This result was supported by the highest loadings of BADGE and derivatives the highest scores of experiments conducted at 336 h on PC1 (55.92% of the total variance). The PC temperature scores and loading plots for the 5% DMSO extraction experiments are presented in Figure 8, in which PC1 explained 73.64% of the total variance and PC2 explained the remaining 26.36%. A comparison of both plots further confirmed that increasing the temperature facilitates the migration of BADGE and chlorinated and hydroxyl derivatives when 5% DMSO is used as a simulant in line with the results for other stimulants.

### 3.2. Discussion of ASCA Modeling of Multi-Layered Composite Material Extracts

Similar to the results of the can lining experiments, the main factors and their interactions were all statistically significant (ref. to Table 2); however, temperature and the interaction between time and temperature accounted for the greatest amount of the total variance.

The migration of BADGE and related compounds in multi-layered composite lining experiments is less pronounced compared to can lining experiments and the compounds not detected in some of simulant extracts are located in the centre of corresponding loadings plots. The reason for centre location of acute toxicity endpoint (Microtox) in Supplementary Figures S3 and S4 is that bioluminescence inhibition for all extracts is 100%.

The PC scores plots for multi-layered composite lining extracts with all simulants demonstrated clear differentiation according to different contact times and temperatures. We here only focus on the most substantial relations between variables and experimental conditions. The ASCA model results for distilled water extraction experiments reflected the relation of BADGE·HCl with experiments conducted at 48 h (Supplementary Figure S3) and at 65 °C (Supplementary Figure S4). The loadings of all BADGE and related compounds suggested that their migration was most likely to occur with shorter contact times of 12 h and 48 h.

By contrast, the ASCA model results for the 5% ethanol extraction experiments (Figures S5 and S6) showed a lack of migration of the BADGE compounds. The temperature scores and loadings plots reflected an increase of endocrine disruption potential (YES+, YES-, YAS-) and acute toxicity with increasing temperature (Supplementary Figure S6).

The ASCA model results for 3% acetic acid extraction experiments (Supplementary Figures S7 and S8) highlighted specific relations between the migration of BADGE compounds and experimental conditions. In particular, the migration of BADGE·H_2_O·HCl occurred with prolonged contact times, since the migration of BADGE·H_2_O and BADGE·2H_2_O occurred at 48 h (Supplementary Figure S7). The PC1 temperature scores demonstrated temperature-dependent variations from 65 °C to 121 °C (Supplementary Figure S8). The positive PC1 temperature loadings of BADGE, BADGE·H_2_O, and BADGE·2H_2_O revealed an increase in the migration of these compounds with increasing temperature. Notably, the group formed by YAS+, BADGE·H_2_O, and BADGE·2H_2_O could be related to experiments conducted at 121 °C.

The most important finding from the ASCA model of the 5% DMSO extraction experiments (Supplementary Figures S9 and S10) is the inverse relation between the migration of BADGE related compounds and temperature. The PC1 (85.13% of the total variance) temperature scores showed dependence from 121 °C to 25 °C (Supplementary Figure S10a), and the positive factor loadings of all investigated compounds indicated their migration at lower temperatures. The presence of BADGE and chlorinated and hydroxyl derivatives at low temperatures was also accompanied by the higher acute toxicity of these extracts.

### 3.3. Discussion of ASCA Modeling of Cup Extracts

The main factors (temperature and contact time) and their interactions were statistically significant for all ASCA models (*p* < 0.01) in line with the models obtained for the can and multilayer composite experiments. Although the majority of the variance for all ASCA models was due to the interaction between time and temperature, the distilled water and 3% acetic acid extracts showed a greater influence of time than temperature, which is the opposite pattern than that obtained for the can and multilayer composite experiments (due to different characteristics of materials used given packaging production).

The PC temperature scores plots (Supplementary Figures S12a, S14a and S16a) distinguished extracts obtained at different temperatures and separated out the extracts subjected to microwave heating specifically. The PC time score plot revealed four groups of distilled water extracts, in which those obtained at 6 h and 12 h formed a common group (Supplementary Figure S11a). The positive PC1 time loadings of BADGE·2H_2_O and BADGE·H_2_O·HCl indicated that their migration occurs at shorter contact times, while the negative loading of BADGE·H_2_O indicated the migration of this compound with prolonged contact times. Considering both the temperature scores and loadings plots (Supplementary Figure S12), enhanced migration of BADGE·HCl, BADGE·2H_2_O, and BADGE·H_2_O·HCl was observed for extract obtained with microwave oven heating. Moreover, all of the endocrine disruption and acute toxicity endpoints showed positive PC1 temperature loadings.

The ASCA model results for the 5% ethanol extraction experiments are presented in Figures S13 and S14. The PC time scores plot indicated three main groups, and PC2 demonstrated a pattern of contact time dependence (Supplementary Figure S13a). The migration of studied compounds occurred at shorter contact times, whereas acute toxicity increased with the increase of contact time. The PC temperature scores and loadings plots demonstrated the enhanced migration of BADGE·2H_2_O and BADGE·H_2_O·HCl in extracts exposed to hotter solvents, which was accompanied by their increased acute toxicity (Supplementary Figure S14).

The ASCA model results for the 3% acetic acid extraction experiments are presented in Supplementary Figures S15 and S16. The PC time scores plot indicated four main groups (Supplementary Figure S15a) in which enhanced migration of BADGE·2H_2_O and BADGE·H_2_O was observed for extracts obtained over prolonged times (Supplementary Figure S15b). In contrast to the results for the distilled water and 5% ethanol experiments, the migration of BADGE and related compounds (BADGE·HCl, BADGE·2H_2_O, and BADGE·H_2_O) occurred for extracts exposed to 25 °C rather than those obtained with a hot solvent or microwave oven treatments (Supplementary Figure S16).

## 4. Materials and Methods

### 4.1. Chemicals

The standards used in the study for instrumental analysis of the extracts of packaging materials were obtained from Sigma-Aldrich (St. Louis, MO, USA), including BADGE (CAS no. 1675-54-3), bisphenol A (3-chloro-2-hydroxypropyl)(2,3-dihydroxypropyl) ether (BADGE·HCl·H_2_O, CAS no. 227947-06-0), bisphenol A (2,3-dihydroxypropyl) glycidyl ether (BADGE·H_2_O, CAS no. 76002-91-0), bisphenol A (3-chloro-2-hydroxypropyl) glycidyl ether (BADGE·HCl, CAS no. 13836-48-1), bisphenol A bis(2,3-dihydroxypropyl) ether (BADGE·2H_2_O, CAS no. 5581-32-8), bisphenol A bis(3-chloro-2-hydroxypropyl) ether (BADGE·2HCl, CAS no. 4809-35-2), BFDGE (CAS no. 2095-03-6), bisphenol F bis(2,3-dihydroxypropyl) ether (BFDGE·H_2_O, CAS no. 72406-26-9), bisphenol F bis(3-chloro-2-hydroxypropyl) ether (BFDGE·2HCl, CAS no. 374772-79-9), and three-ring novolac glycidyl ether (mixture of isomers) (CAS no. 158163-01-0). The internal standard (IS) d_10_-labeled BADGE (CAS no. 1675-54-3) was supplied by Cambridge Isotope Laboratories Inc. (Cambridge, UK). Methanol (MeOH, CAS no. 67-56-1), acetonitrile (ACN, CAS no. 75-05-8), ethyl acetate (EtOAc, CAS no. 141-78-6), dichloromethane (DCM, CAS 75-09-2), and acetone (CAS no. 67-64-1) were of LC-MS-hypergrade purity and obtained from Merck KGaA (Darmstadt, Germany). DMSO (CAS no. 67-68-5) and ammonium formate (CAS no. 540-69-2) were purchased from Sigma-Aldrich. All reagents were of analytical purity-grade. Ultrapure water was produced by the Milli-Q Gradient A10 system equipped with an EDS-Pak cartridge (Merck-Millipore, Darmstadt, Germany). The SPE disposable cartridges (Chromabond C_18_, 500 mg, 6 mL) were supplied by Sigma-Aldrich, and the Strata-X column (C_18_, 500 mg, 6 mL) was obtained from Shim-Pol (Warsaw, Poland).

The following chemicals were used for preparing the simulation media: sodium chloride (CAS no. 7440-23-5, Sigma Aldrich, Germany), dipotassium phosphate (CAS no. 7758-11-4, Ciech S.A., Poland), calcium chloride (CAS no. 7440-70-2, Eurochem BGD, Poland), magnesium chloride (CAS no. 7786-30-3), potassium chloride (CAS no. 7440-09-7), potassium carbonate (CAS no. 584-08-7), lactic acid (CAS no. 79-33-4), urea (CAS no. 57-13-6) (all from POCH S.A., Poland), ammonium hydroxide (25% *w*/*w*) (CAS no. 1336-21-6), acetic acid (35–38% *w*/*w*, CAS no. 64-19-7) (Chempur, Poland), distilled water, and EDC-Pak cartridge (Merck, Germany).

The Microtox^®^ kit (2% NaCl, lyophilized *Vibrio fischeri* bacteria, Microtox Diluent, Microtox Acute Reagent, Osmotic Adjusting Solution (OAS), and Reconstitution Solution (RS)) was purchased from Modern Water Ltd. (Cambridge, UK). All reagents were of analytical grade or higher (reagents used for microbiological purposes). Reagents used for the XenoScreen YES/YAS assay were purchased from Xenometrics G. A. (Allschwil, Switzerland), including vials containing hERα yeast cells (for the YES assay) and hAR *Saccharomyces cerevisiae* yeast cells (for the YAS assay) on a filter paper, basal medium, vitamins, l-aspartic acid (CAS no.56-84-8), l-threonine (CAS no. 72-19-85), copper sulfate solutions (CAS no. 7758-98-7), and CPRG (chlorophenol red-β-D-galactopyranoside) (CAS no. 99792-79-9), and vials with 17β-estradiol (CAS no. 50–28-2), 5α-dihydrotestosterone (CAS no. 521-18-6), 4-hydroxytamoxifen (CAS no. 68392-35-8), flutamide (CAS no. 13311-84-7), and DMSO (CAS no. 67-68-5). The 96-well plates, gas-permeable plate sealers, and culture flasks with a gas-permeable filter cap were purchased from GenoPlast Biochemicals (Poland). All reagents were of analytical-grade purity or better in the case of reagents used for microbiological purposes.

Electronic multi- and single-channel pipettes were from Eppendorf (Germany). NaOH (CAS no. 1310-73-2) and HCl (CAS no. 7647-01-0) were purchased from Avantor Performance Materials S.A. (Poland). The CP411 pH-meter was from Metron (Warsaw, Poland). The Thermicon P^®^ heater (type K1253S) was from Heraeus Instruments (Heraeus, Germany), and the microwave heating device (Samsung ME 733 K; maximum power 1150 W) and shaker-type water bath 357 were from Elpin Laboratory Instruments (Lubawa, Poland).

Artificial saliva was prepared in accordance with the guidelines described in the DIN: 53160-1:2010-10 standard [9], comprising 0.53 g/dm^3^ NaCl, 0.33 g/dm^3^ KCl, 0.15 g/dm^3^ CaCl_2_·2H_2_O, 0.76 g/dm^3^ K_2_HPO_4_·3H_2_O, 0.17 g/dm^3^ MgCl_2_·6H_2_O, 0.53 g/dm^3^ K_2_CO_3_, and 0.75 g/dm^3^ 1% HCl. The pH of the solution was adjusted to 6.8 using a 1% NH_3_ solution. To minimize background endocrine-disrupting compound contamination, the Milli-Q water was additionally purified with an EDC-Pak cartridge at the stage of preparation of simulation liquids. In addition, to reduce the risk of contamination of the glassware with organics, an additional step of heating the utensils at 450 °C for at least 4 h was applied [10]. The simulation liquids were stored at 4 °C prior to performing the extraction process.

### 4.2. Instrumental and Biological Studies

Owing to the substantial amount of instrumental and biological methodologies used in this study, the details of the methods for chemometric data evaluation are presented in the electronic supplement, and the main procedures are also described in Szczepańska et al. [11,12].

Because of substantial amount of instrumental and biological methodologies used and applied in the research these data are presented in electronic supplement to facilitate readers with details of instrumental and biological approach used to extract data necessary for chemometric data evaluation (although the extraction procedures and biological methods are already given in Szczepańska et. al. [11]).

### 4.3. Chemometric Data Analysis

ASCA is a method that is well suited for handling multivariate datasets obtained by a controlled experimental design [13,14]. ASCA combines analysis of variance with principal components analysis (PCA) to assess and interpret the variation induced by experimental factors. The ASCA model for a study of two factors, time, and temperature in the present study could be formulated according to Equation (1) as follows:

(1) $$X=\mu +X_{time}+X_{temperature}+X_{time\times temperature}+E,$$

where X is the total variance, µ is the mean of X, $X_{time}$ and $X_{temperature}$ are the variances associated with the main factors, $X_{time\times temperature}$ is the variance related to the interaction of the two factors, and E is the residual variance. The SCA of factors and interaction matrices was performed according to their PCA decomposition on scores ($T_{i}$) and loadings ($P_{i}$) as shown in Equation (2):

(2) $$X=\mu +T_{time}P_{time}^{T}+T_{temp}P_{temperature}^{T}+T_{time\times temperature}P_{time\times temperature}^{T}+E,$$

The scores reflect the distribution of experiments obtained under different experimental conditions since the loadings indicate the relationship between independent variables (i.e., endocrine disruption endpoints, acute toxicity endpoint, and BADGE and its derivatives concentrations) and the effects linked to the particular factor. In line with previous ASCA applications, a maximum of two components per effect matrix is a reasonable limit for interpretation [15]. The statistical significance of the effects of each factor included in the ASCA was then evaluated by a permutation test (i.e., 10,000 permutations) as thoroughly described by Timmerman et al. [15] (significance of the effects is presented by the *p*-values in Table 1, Table 2 and Table 3). Before the analysis, the input data (acute toxicity, endocrine potential, concentrations of BADGE and related compounds) were autoscaled. All ASCA modeling calculations were performed in MATLAB R2018b (Mathworks Inc., Natick, MA, USA) using the PLS Toolbox 8.7 software package (Eigenvector Research Inc., Manson, WA, USA).

## 5. Conclusions

Food and its packaging materials have received increasing research attention given their enormous impact on food quality, and ultimately food stability, for both short- and long-term storage. Social awareness of the potential migration of trace pollutants from packaging materials into the contained food products (and other goods) is already increasing and is expected to grow further in the coming decades. Accordingly, novel integrated approaches and methodologies are needed to best describe these phenomena in a holistic manner utilizing biological, instrumental, and chemometric tools. The present work provides one such holistic approach to explain the observed acute toxicity and endocrine potential according to measured indices of trace organic substances that are present in extracts exposed to different simulation media under different conditions.

The ASCA results for the three types of packaging materials analyzed (can lining, multi-layered composite material, and cup surface) in various simulation media pointed to the common significant influence of contact time and temperature on the toxicity, endocrine potential, and migration of BADGE and its derivative compounds into the extracts. Except for the 5% DMSO extracts, the partitioning of total variance for the can and multi-layered composite material experiments indicated a stronger influence of temperature and the interaction between time and temperature compared to the effect of contact time. By contrast, for the cup extracts obtained with distilled water and 3% acetic acid as the simulation media, the time factor explained a higher fraction of the total variance than temperature. The dominant role of temperature on the migration of BADGE and related compounds in the can lining experiments was confirmed by the increase of their migration with increasing temperature in all simulation media. However, the positive relationship between BADGE and related compound migration and temperature was less pronounced for the multi-layered composite materials, and the migration of the 5% DMSO extracts showed the opposite pattern, occurring at lower temperatures.

The migration of BADGE and its derivatives compounds in the cup extractions largely depended on the simulation medium used. The enhanced migration of BADGE and related compounds for distilled water extracts was observed with microwave oven treatment, whereas greater migration was observed with hot solvents for the 5% ethanol extractions and under room temperature (here 25 °C) for the 3% acetic acid extracts. There was no clear pattern of studied compounds migration with respect to the contact time. The migration of analytes in the 5% DMSO can lining extracts occurred with prolonged time, whereas the distilled water extracts of the multi-layered composite material and 5% ethanol cup extracts showed migration at shorter contact times. There was no stable relation observed between the endocrine disruption potential and acute toxicity of the extracts with the amount of monitored compounds that migrated for all investigated packages. Thus, further investigations are needed to determine the source of the acute toxicity and endocrine disruption potential for extracts in which these effects could not be attributed to the migration of BADGE and related compounds.

In all assessments, time, temperature, and their interactions were significant for all models, although the effects of temperature and the interaction were more dominant overall than those of time. There was also a general trend of increased migration of studied compounds with increased temperature, although this pattern was also dependent on the experimental conditions. This approach clearly demonstrated the interactions and grouping of these different factors to highlight the factors contributing to quality deterioration and potential toxicity or leakage into food items. We believe that our study makes a significant contribution to the literature given increasing public awareness of the potential risks of packaging materials on their health due to phenomena occurring with contact with the goods contained within; therefore, more complex and standardized methods of quality assurance and assessment are needed beyond traditional single-level instrumental analysis. Our findings demonstrate the value of using a holistic approach to best partition the effects contributing to the end points of these assessments, and broadly speaking, offer further guidance for adopting such a methodology. These results can therefore serve as a useful reference for understanding the phenomena related to the impacts of food packaging materials on quality for long- and short-term storage, while offering a general method for analysis.

## Figures and Tables

**Table 1:** ASCA results for can lining extracts in different simulants: significance and portioning of the total variance into individual terms corresponding to the factors and their interactions.

Solvent	Factor	Expl. Var. [%]	Significance (*p*-Value)
Distilled water	time	14.7	< 0.01
	temperature	55.3	< 0.01
	time × temperature	27.1	< 0.01
	residuals	2.8	
5% ethanol	time	17.4	< 0.01
	temperature	31.6	< 0.01
	time × temperature	44.9	< 0.01
	residuals	6.1	
3% acetic acid	time	29.0	< 0.01
	temperature	30.4	< 0.01
	time × temperature	38.8	< 0.01
	residuals	1.7	
5% DMSO	time	26.1	< 0.01
	temperature	25.5	< 0.01
	time × temperature	46.2	< 0.01
	residuals	2.2	

**Table 2:** ASCA results for multi-layer composite material extracts in different simulants: significance and portioning of the total variance into individual terms corresponding to the factors and their interactions.

Solvent	Factor	Expl. Var. (%)	Significance (*p*-Value)
Distilled water	time	13.4	< 0.01
	temperature	26.4	< 0.01
	time × temperature	49.8	< 0.01
	residuals	10.4	
5% ethanol	time	12.7	< 0.01
	temperature	48.0	< 0.01
	time × temperature	34.1	< 0.01
	residuals	5.2	
3% acetic acid	time	27.6	< 0.01
	temperature	28.0	< 0.01
	time × temperature	41.6	< 0.01
	residuals	2.8	
5% DMSO	time	17.0	< 0.01
	temperature	37.8	< 0.01
	time × temperature	42.2	< 0.01
	residuals	3.0	

**Table 3:** ASCA results for cup internal surface extracts in different simulants: significance and partitioning of the total variance into individual terms corresponding to the factors and their interaction.

Solvent	Factor	Expl. Var. [%]	Significance (*p*-Value)
Distilled water	time	26.1	< 0.01
	temperature	14.2	< 0.01
	time × temperature	54.2	< 0.01
	residuals	5.5	
5% ethanol	time	23.9	< 0.01
	temperature	33.1	< 0.01
	time × temperature	36.0	< 0.01
	residuals	7.0	
3% acetic acid	time	26.4	< 0.01
	temperature	21.9	< 0.01
	time × temperature	46.4	< 0.01
	residuals	5.2	

## Supplementary Materials

The following are available online, Figure S1: Presentation of recovery values obtained with STRATA X and Chromabond extraction columns. Figure S2: Comparison of extraction efficiencies with different solvents. Table S1: The recovery rates obtained during instrumental method validation. Figure S3: SCA „time” scores (**a**) and loadings (**b**) plot for distilled water extraction experiments. Figure S4: SCA „temperature” scores (**a**) and loadings (**b**) plot for distilled water extraction experiments. Figure S5: SCA „time” scores (**a**) and loadings (**b**) plot for 5% ethanol extraction experiments. Figure S6: SCA „temperature” scores (**a**) and loadings (**b**) plot for 5% ethanol extraction experiments. Figure S7: SCA „time” scores (**a**) and loadings (**b**) plot for 3% acetic acid extraction experiments. Figure S8: SCA „temperature” scores (**a**) and loadings (**b**) plot for 3% acetic acid extraction experiments. Figure S9: SCA „time” scores (**a**) and loadings (**b**) plot for 5% DMSO extraction experiments. Figure S10: SCA „temperature” scores (**a**) and loadings (**b**) plot for 5% DMSO extraction experiments. Figure S11: SCA „time” scores (**a**) and loadings (**b**) plot for distilled water extraction experiments. Figure S12: SCA „temperature” scores (**a**) and loadings (**b**) plot for distilled water extraction experiments. Figure S13: SCA „time” scores (**a**) and loadings (**b**) plot for 5% ethanol extraction experiments. Figure S14: SCA „temperature” scores (**a**) and loadings (**b**) plot for 5% ethanol extraction experiments. Figure S15: SCA „time” scores (**a**) and loadings (**b**) plot for 3% acetic acid extraction experiments. Figure S16: SCA „temperature” scores (**a**) and loadings (**b**) plot for 3% acetic acid extraction experiments.
