# Supplementary material for: Influence of Storage Time and Temperature on the Toxicity, Endocrine Potential, and Migration of Epoxy Resin Precursors in Extracts of Food Packaging Materials
Source: Molecules. 2019 Dec 2;24(23):4396. doi: 10.3390/molecules24234396 (PMC6930607; doi:10.3390/molecules24234396)
Supplement: Supplementary file 1 [file molecules-24-04396-s001.pdf]

Influence of storage time and temperature on the toxicity, endocrine potential, and migration of epoxy resin precursors in extracts of food packaging materials

Błażej Kudłak[1#], Natalia Jatkowska[1*], Paweł Kubica[1], Galina Yotova[2], Stefan Tsakovski[2]

[1] Gdańsk University of Technology, Faculty of Chemistry, Department of Analytical Chemistry, 11/12 Naturowicza, 80-952 Gdańsk, Poland
[2] Sofia University “St. Kliment Ohridski”, Faculty of Chemistry and Pharmacy, Chair of Analytical Chemistry, 1164 Sofia, Bulgaria
*Corresponding Author: natalia.szczepanska@pg.edu.pl, ORCID: 0000-0001-8268-3287

[#]ORCID: 0000-0002-2237-2927

2. Results and discussion

2.1. ASCA modelling of cans extracts

Results and their discussion are presented in the main text.

2.2. ASCA modelling of multi-layered composite material extracts

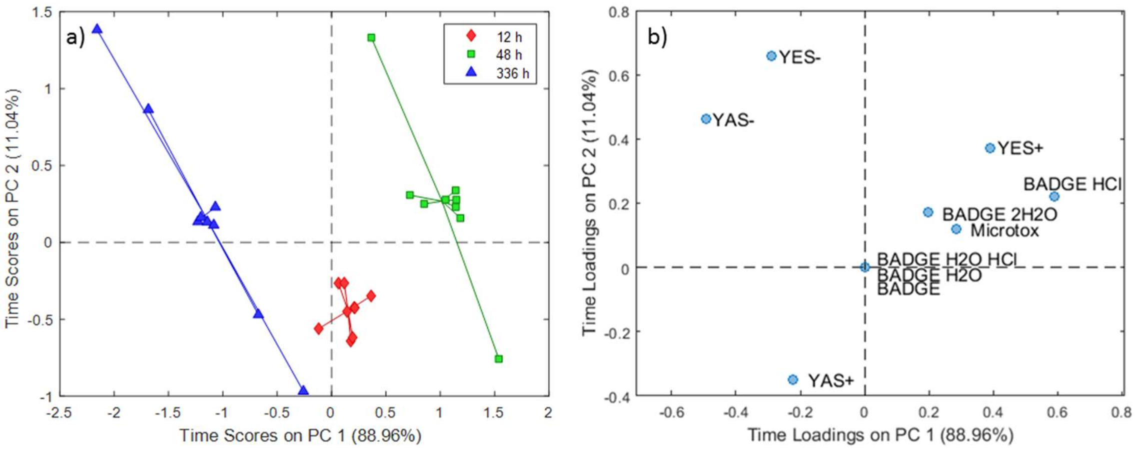

Suppl. Fig. 3. SCA „time” scores (a) and loadings (b) plot for water extraction experiments

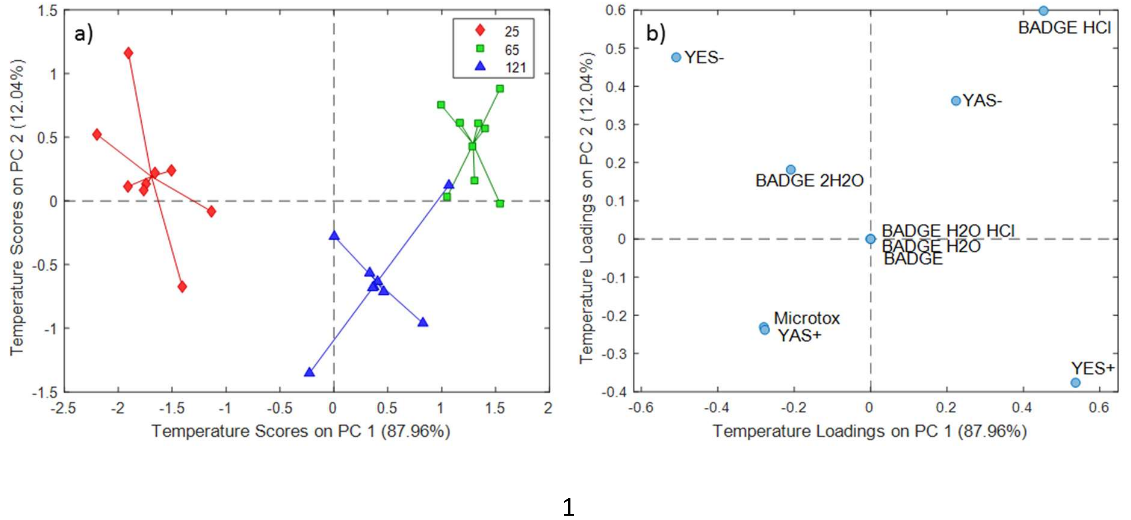

Suppl. Fig. 4. SCA „temperature” scores (a) and loadings (b) plot for water extraction experiments

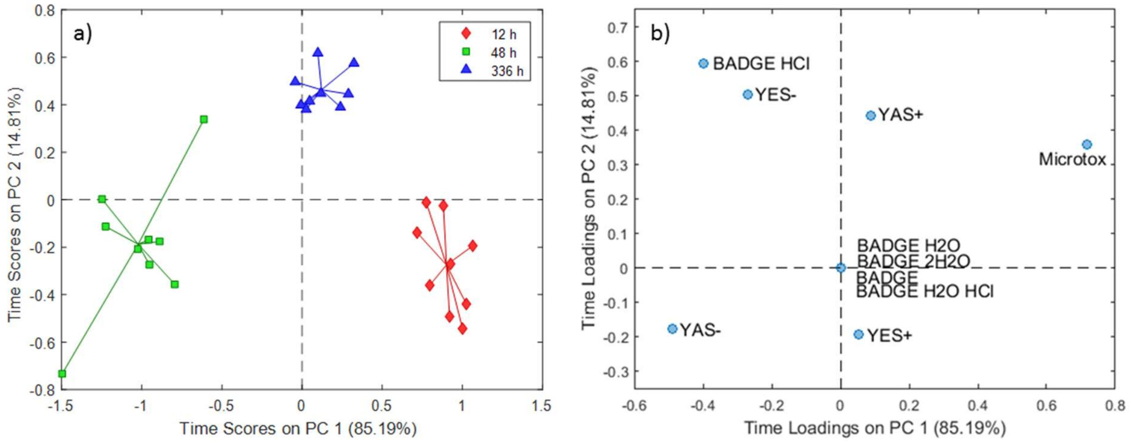

Suppl. Fig. 5. SCA „time” scores (a) and loadings (b) plot for 5% ethanol extraction experiments

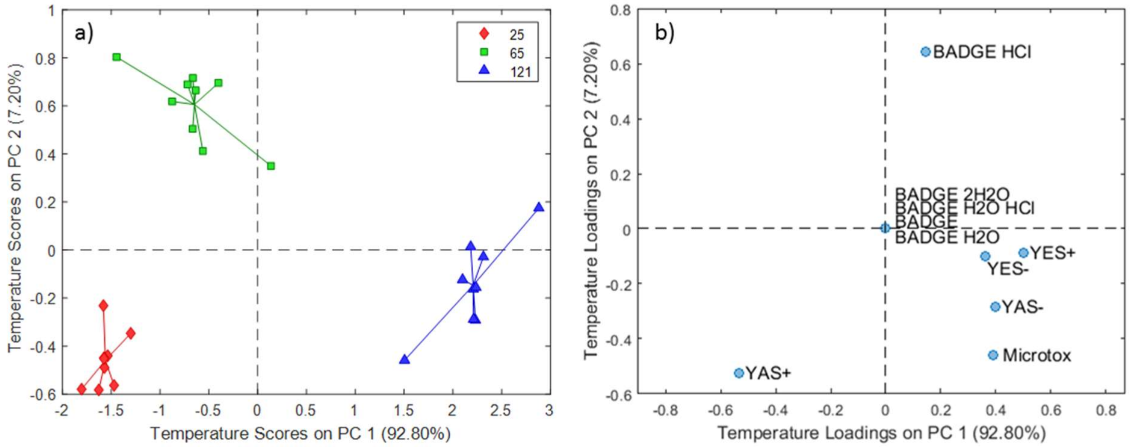

Suppl. Fig. 6. SCA „temperature” scores (a) and loadings (b) plot for 5% ethanol extraction experiments

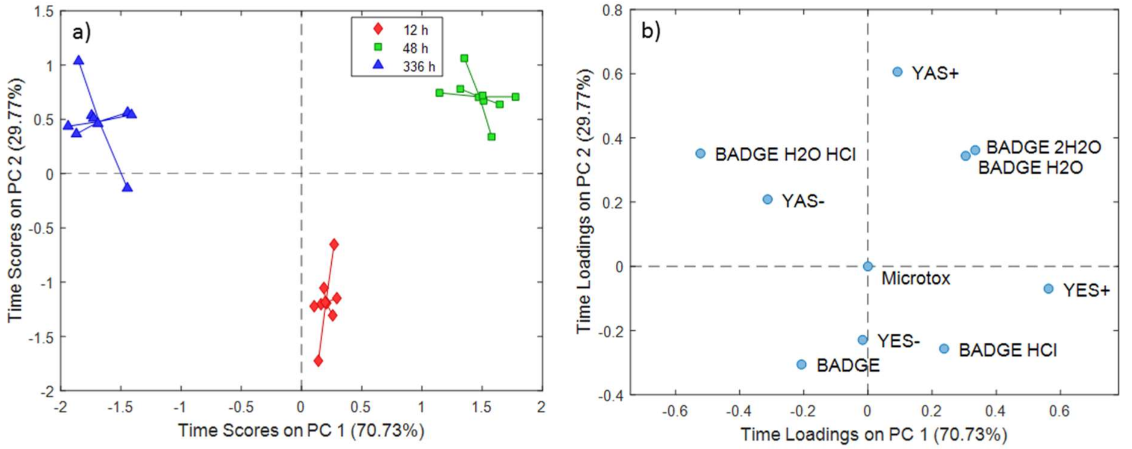

Suppl. Fig. 7. SCA „time” scores (a) and loadings (b) plot for acetic acid extraction experiments

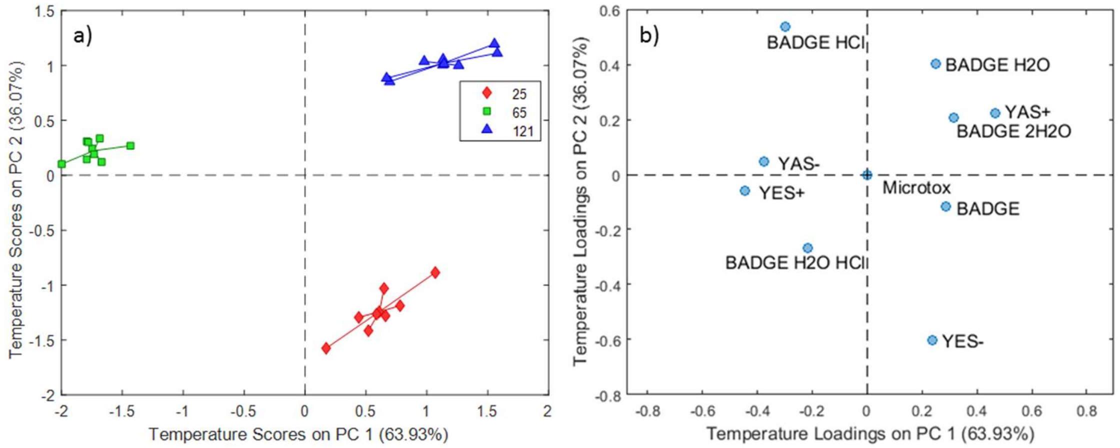

Suppl. Fig. 8. SCA „temperature" scores (a) and loadings (b) plot for acetic acid extraction experiments

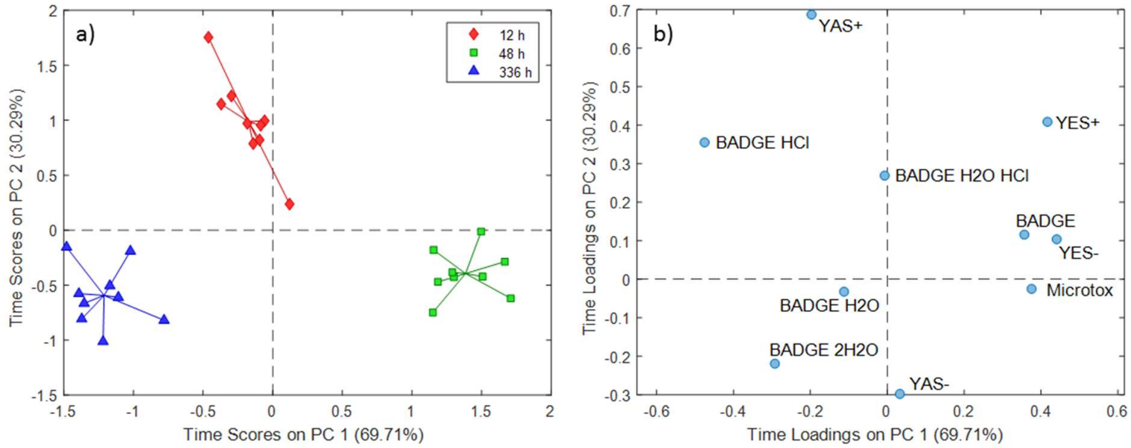

Suppl. Fig. 9. SCA „time" scores (a) and loadings (b) plot for 5% DMSO extraction experiments

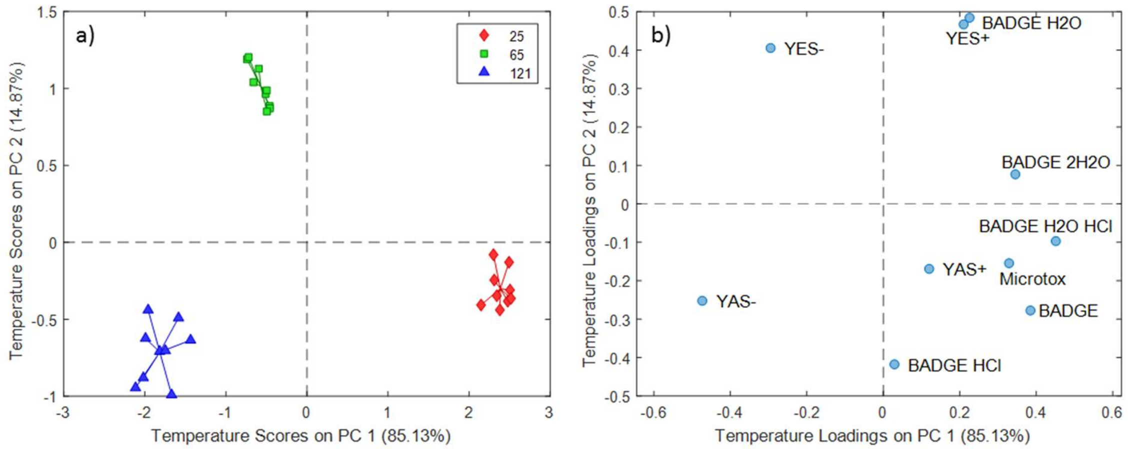

Suppl. Fig. 10. SCA „temperature" scores (a) and loadings (b) plot for 5% DMSO extraction experiments

2.3. ASCA modelling of cups extracts

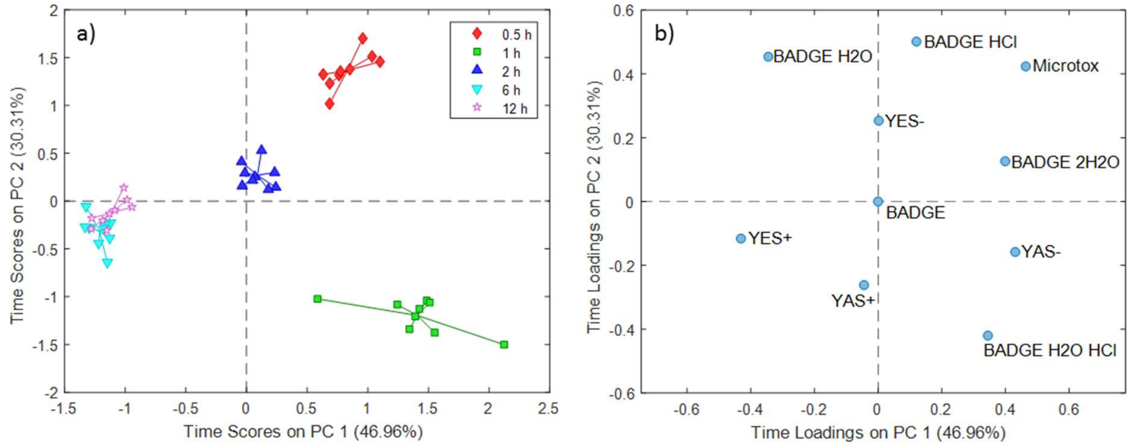

Suppl. Fig. 11. SCA „time" scores (a) and loadings (b) plot for water extraction experiments

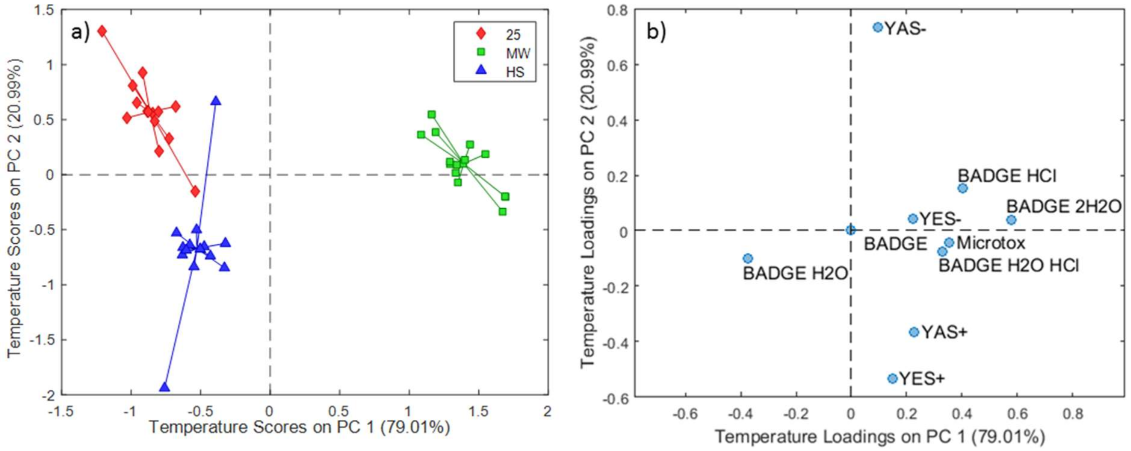

Suppl. Fig. 12. SCA „temperature" scores (a) and loadings (b) plot for water extraction experiments

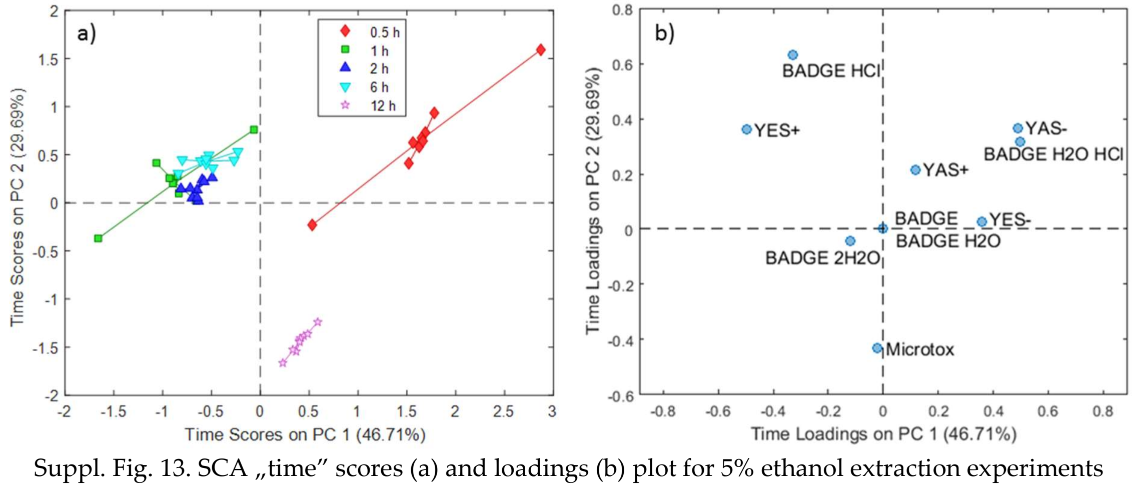

Suppl. Fig. 13. SCA „time" scores (a) and loadings (b) plot for 5% ethanol extraction experiments

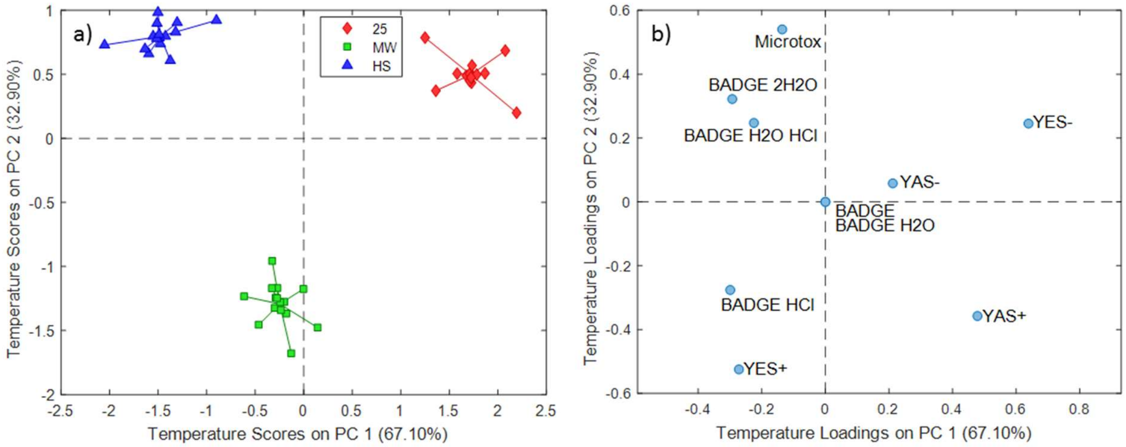

Suppl. Fig. 14. SCA „temperature" scores (a) and loadings (b) plot for 5% ethanol extraction experiments

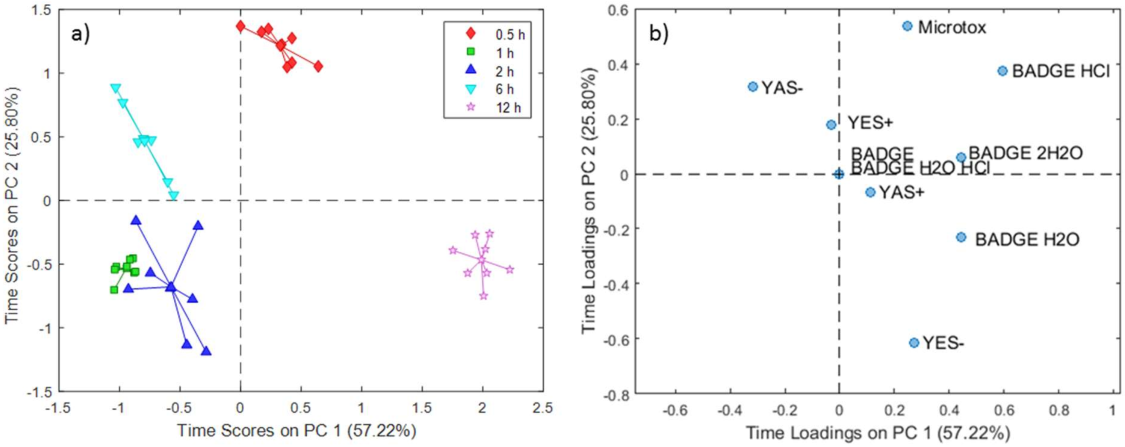

Suppl. Fig. 15. SCA „time" scores (a) and loadings (b) plot for 3% acetic acid extraction experiments

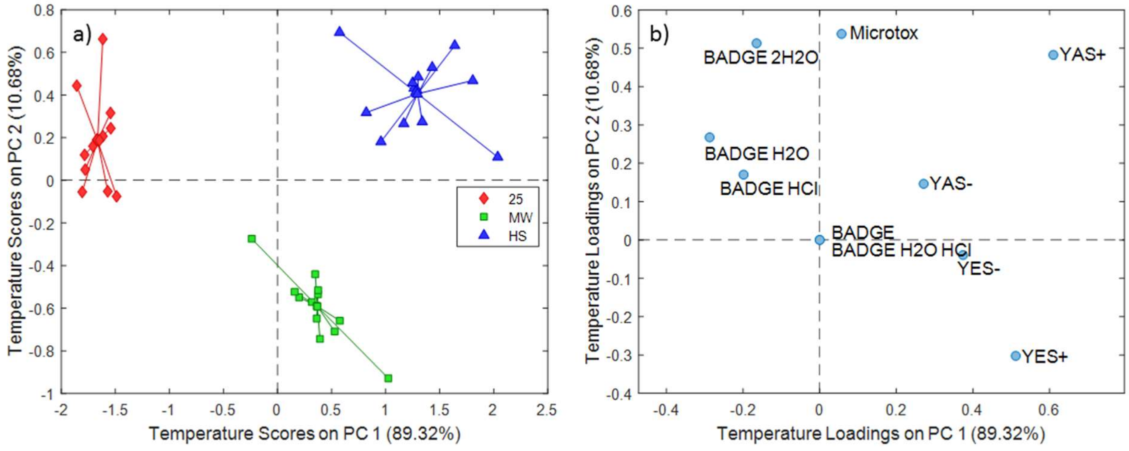

Suppl. Fig. 16. SCA „temperature" scores (a) and loadings (b) plot for 3% acetic acid extraction experiments

4. Materials and methods

4.1. Chemicals

Given in main text.

4.2. Instrumental and biological studies

Despite the fact that, details of chromatographic analysis are presented in Szczepańska et al. [11], in order to clarity, below basic information on the chromatographic separation are presented. Chromatographic separation was carried out using the ultra-performance liquid chromatography (UPLC) Nexera X2 system (Shimadzu, Japan), which consisted of a DGU-20A5R degasser, CBM-20A controller, LC-30AD binary pump, SIL-30AC autosampler, and CTO-20AC column oven. The separation was achieved using Kinetex® XB-C8 column (100 × 2.1 mm, 2.6 μm in core-shell technology). The column oven temperature was set to 45°C, the flow rate was kept at 1.0 mL/min, and the injection volume was set to 2.0 μL. The mobile phase used for the separation was 40 mM ammonium formate (component A) and MeOH (component B). The chromatographic separation was performed in gradient elution mode: 0 min (35% B), 7 min (85% B), and 9 min (85% B). After each analysis, the initial column conditions were restored over 5 min.

4.2.1. Preparation of standards solutions

Individual stock solutions (approximately 0.5 mg/mL) of all analytes were prepared separately by dissolving appropriate amounts of analytical standards in MeOH. The working solution for calibration was obtained by mixing the stock solutions and diluting them with MeOH. All solutions were stored at -20 °C. BADGE d10 was used as the IS and prepared separately by dissolving a proper amount to obtain a stock solution at a concentration of 2.5 μg/mL.

4.2.2. Chromatographic conditions

Details of chromatographic analyses are presented in Szczepańska et. al. [12], and the basic information is outlined here in the electronic supplementary material.

4.2.3. Samples, sample preparation and spiking procedures

Details of simulation liquids preparation and sample collection are presented in Szczepańska et al. [11]. Before loading the sample, the SPE cartridges were conditioned with 6 mL of MeOH and 6 mL of water with a flow rate of approximately 3 mL/min. Next, 15 mL of the sample was loaded at the same flow rate. After loading, the cartridge was kept under vacuum for 20 min to remove residual water. Finally, the analytes were eluted using 10 mL of MeOH. The obtained extract was collected in a test tube and evaporated to dryness at 45 °C under a gentle stream of nitrogen. One millilitre of MeOH was added to dissolve the residues prior to chromatographic injection.

Owing to the absence of certified materials, it was necessary to prepare spiked samples. Fortified samples were prepared using separate simulant liquids as matrices, including distilled water, 3% acetic acid, 5% ethanol, 5% DMSO, and artificial saliva solution. Analytes were added to each simulant liquid (50 mL, in triplicate) to obtain different concentration of a particular analyte (0.0033, 0.0067, 0.033, 0.066, 0.17, 0.33, 0.66, 1.67, 3.33, 6.67, 13.33, 33.33, 66.67 ng/mL). These spiked samples were then used to construct two calibration curves in the range of 0.0033–0.33 ng/mL and 0.33–66.67 ng/mL, respectively, to obtain a linear response of each analyte over a wide concentration range. For each sample, the IS concentration was fixed at 1.67 ng/mL. Samples prepared for evaluation of recoveries and repeatability determination were prepared in the same manner. The recoveries were measured by

extracting analytes from 2×15 mL of each simulant liquid spiked with the analytes at three concentration levels for each studied range (0.033, 0.17, 0.67 and 3.33, 6.66, 33.33 ng/mL, respectively). Fresh matrix matched samples used for calibration were prepared for every batch of samples.

4.2.4. Extraction (for instrumental studies) conditions

To optimize the extraction process, a series of experiments were carried out using the 3% acetic acid solution spiked at 3.33 ng/mL. Each experiment was carried out in triplicate.

Type of sorbent

To determine the optimal sorbent type, the retention stability of Strata-X (a sorbent based on a surface-modified styrene-divinylbenzene polymer) and the more commonly used Si-C18 (Chromabond) were compared. As shown in Supplementary Fig. 1, the recoveries obtained with Chromabond were higher for most of the target compounds, ranging from 94% for 3-ring NOGE to 77% for BADGE·H2O·HCl, with a coefficient of variation (CV) of 1.5–7.2%, whereas the recoveries with Strata-X were 44– 92%. Therefore, Si-C18 was selected as the sorbent for further analysis.

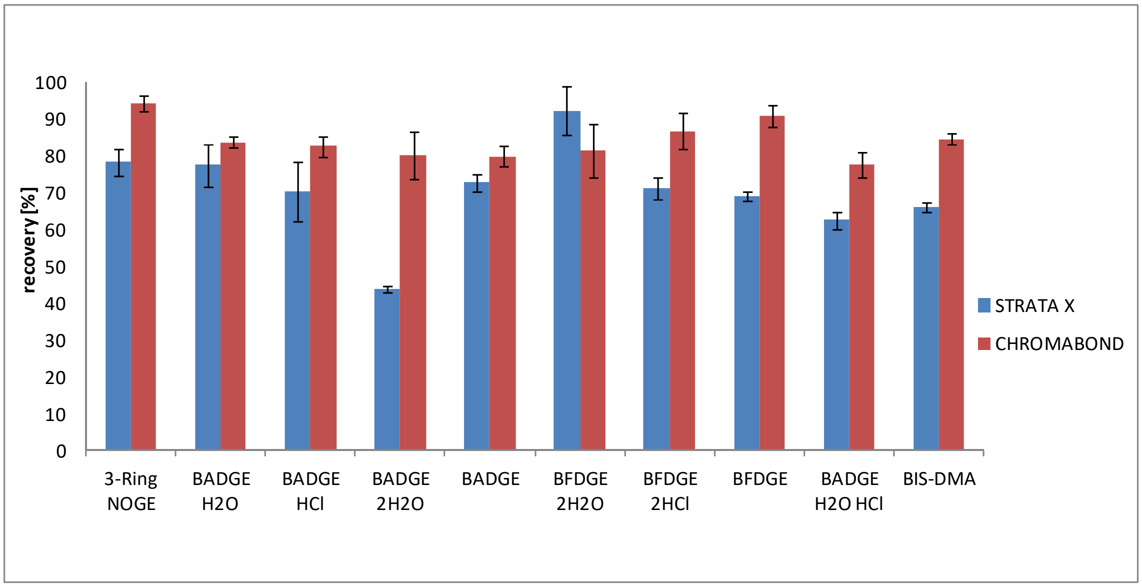

Supplementary Figure 1. Presentation of recovery values obtained with STRATA X and Chromabond extraction columns (n=3).

Elution solvent and its volume

We next determined the optimal elution solvent type and volume, which is a crucial step to achieve good extraction efficiency. We compared the eluting efficiency of MeOH, ACN, EtOAc:DCM (50:50), and acetone: ACN (50:50) as potential solvents. As shown in Supplementary Fig. 2, MeOH was found to be a more effective extraction solvent compared to others and was selected for further analysis. The influence of the volume of MeOH was tested at 5, 7.5, 10, and 15 mL. The highest recovery values of most analytes were obtained using 10 mL and 15 mL MeOH, but there was no significant difference between these two volumes. Therefore, 10 mL was selected as the optimum volume of the extraction solvent.

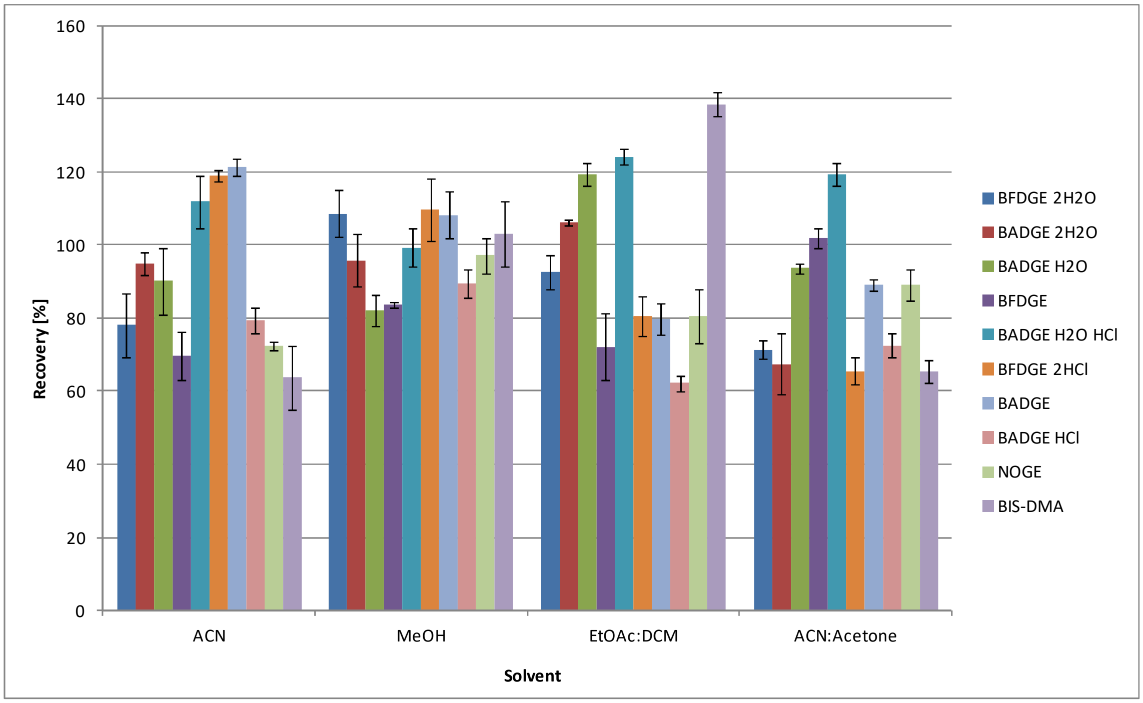

Supplementary Figure 2. Comparison of extraction efficiencies with different solvents

4.2.5. Method validation

The performance of the analytical method was evaluated by determination of the limit of detection (LOD) and limit of quantitation (LOQ), recoveries, and accuracy. Unspiked blank samples of simulation liquids were prepared and analyzed. After BADGE·2H_2O, BADGE·H_2O·HCl, and 3-ring NOGE peaks were detected, appropriate corrections were made in the analysis of real samples and spiked samples. The calibration curves were linear in the studied concentration range (0.0033–1.67 ng/mL and 1.67 –66.67 ng/mL). The determination coefficients (R^2) for all analytes were greater than 0.990. The weighting factor 1/x was applied to every calibration curve to increase the accuracy at the lowest concentration range. Quantification and validation were performed with a matrix-matched calibration curve. The LOD values were calculated using the following formula: LOD = 3.3 × S_b/a, where S_b is the standard deviation of the intercept of the calibration curve, and a is the slope of the calibration curve. The LOQ value was calculated by multiplying the LOD value by 3. LOQ values were found to be 0.07–0.09. The recoveries of the proposed method were measured by extracting the analytes under optimized conditions with the SPE method from spiked samples at six concentrations (0.033, 0.17, 0.67, 3.33, 6.67, and 33.33 ng/mL, n=3). As shown in Supplementary Table 1 the obtained results were satisfactory in terms of precision and repeatability, which indicate that developed method is suitable for determination of BADGE related compounds in the stimulant liquid samples.

Supplementary Table 1. The recovery rates obtained during instrumental method validation

matrix	analyte	LOD [ng/mL]	LOQ [ng/mL]	Mean recovery [%] (RSD)					
				0.03 [ng/mL]	0.16 [ng/mL]	0.66 [ng/mL]	3.33 [ng/mL]	6.66 [ng/mL]	33.33 [ng/mL]
water	BFDGE·2H_2O	0.03	0.09	108.11 (4.59)	100.49 (1.65)	104.27 (2.36)	101.42 (1.15)	92.77 (0.83)	116.86 (0.32)
	BADGE·2H_2O	0.03	0.08	109.00(6.92)	107.79 (2.83)	106.80 (6.51)	91.30 (1.45)	98.12 (0.99)	101.12 (0.86)
	BADGE·H_2O	0.03	0.08	113.09 (5.35)	108.16 (2.33)	108.63 (1.17)	105.79 (5.66)	92.96 (2.54)	95.47 (3.80)
	BFDGE	0.03	0.08	108.23 (0.51)	100.94 (6.98)	108.24 (5.95)	95.75 (0.99)	103.26 (5.34)	107.15 (2.22)
	BADGE·H_2O·HCl	0.02	0.07	107.39 (4.88)	108.64 (4.80)	108.93 (1.33)	100.79 (1.73)	92.18 (1.03)	90.97 (1.90)
	BFDGE·2HCl	0.03	0.08	105.28 (2.82)	112.20 (2.74)	103.14 (1.62)	105.23 (4.48)	95.48 (1.61)	108.91 (0.42)
	BADGE	0.02	0.07	107.28 (0.75)	103.94 (4.89)	99.27 (7.14)	96.84 (1.01)	95.53 (0.61)	101.48 (0.15)
	BADGE·HCl	0.02	0.07	107.20 (7.93)	97.48 (2.97)	107.77 (3.23)	103.74 (1.34)	99.20 (0.75)	106.86 (0.84)
	3-Ring NOGE	0.02	0.05	115.75 (0.71)	110.73 (4.67)	108.67 (6.46)	109.72 (1.32)	104.66 (3.80)	108.21 (2.33)
	BIS-DMA	0.03	0.08	110.23 (3.16)	92.10 (1.57)	90.70 (2.10)	101.69 (1.05)	108.45 (0.89)	104.32 (0.88)
3% acetic acid solution	BFDGE·2H_2O	0.03	0.08	99.82 (4.35)	95.20 (0.69)	104.61 (3.64)	109.48 (4.32)	109.78 (0.10)	105.46 (8.71)
	BADGE·2H_2O	0.03	0.08	101.21 (4.95)	98.84 (0.64)	106.96 (1.35)	107.40 (7.50)	99.06 (3.20)	108.14 (5.20)
	BADGE·H_2O	0.02	0.07	93.45 (2.73)	93.52 (3.52)	91.41 (0.91)	90.48 (4.65)	106.08 (9.55)	108.06 (0.76)
	BFDGE	0.02	0.08	98.70 (5.82)	100.41 (3.31)	92.01 (2.00)	92.12 (2.96)	97.02 (4.43)	90.41 (2.72)
	BADGE·H_2O·HCl	0.03	0.08	101.88 (3.94)	102.42 (1.69)	98.87 (6.47)	96.79 (2.27)	99.08 (2.61)	109.91 (1.67)
	BFDGE·2HCl	0.03	0.08	108.88 (6.65)	93.40 (2.00)	109.83 (6.85)	94.35 (5.93)	97.72 (5.77)	107.15 (7.46)
	BADGE	0.02	0.07	111.18 (1.11)	99.78 (4.02)	93.05 (1.56)	95.15 (2.58)	96.23 (2.53)	101.78 (2.81)
	BADGE·HCl	0.03	0.08	106.46 (7.33)	105.61 (5.32)	98.84 (5.93)	106.05 (3.87)	102.34 (3.21)	109.93 (2.03)
	3-Ring NOGE	0.02	0.07	109.35 (4.16)	104.58 (4.48)	100.64 (4.77)	98.46 (2.34)	86.45 (3.01)	94.06 (7.22)
	BIS-DMA	0.03	0.08	109.12 (4.62)	104.35 (0.88)	98.10 (3.61)	100.66 (2.36)	92.80 (1.23)	92.09 (3.08)
5% DMSO solution	BFDGE·2H_2O	0.03	0.08	99.24 (3.93)	104.48 (1.07)	95.12 (4.20)	113.88 (1.65)	114.69 (2.45)	113.43 (1.16)
	BADGE·2H_2O	0.02	0.07	100.44 (1.84)	111.99 (0.28)	100.67 (1.30)	92.46 (6.01)	92.53 (3.79)	90.61 (0.55)
	BADGE·H_2O	0.03	0.08	91.08 (1.23)	91.58 (2.10)	97.00 (4.39)	106.33 (7.01)	90.73 (4.50)	90.35 (3.21)
	BFDGE	0.03	0.08	97.94 (9.69)	98.61 (1.36)	94.79 (0.17)	95.48 (0.22)	92.30 (5.55)	100.87 (1.28)
	BADGE·H_2O·HCl	0.03	0.07	98.47 (8.47)	95.08 (1.70)	88.13 (2.18)	99.15 (8.44)	11.63 (1.85)	110.41 (7.70)
	BFDGE·2HCl	0.03	0.07	94.19 (9.24)	101.45 (4.36)	101.92 (3.45)	94.67 (3.71)	98.67 (3.56)	102.00 (6.86)
	BADGE	0.03	0.06	108.31 (6.72)	90.87 (2.33)	94.09 (5.69)	91.61 (1.77)	96.02 (2.44)	104.18 (0.92)
	BADGE·HCl	0.03	0.08	106.57 (1.38)	93.56 (4.59)	95.08 (1.11)	97.02 (8.01)	96.61 (6.82)	94.42 (2.40)
	3-Ring NOGE	0.03	0.07	105.42 (3.82)	106.40 (0.71)	100.11 (2.15)	108.16 (1.29)	112.48 (5.11)	110.57 (5.17)

	BIS-DMA	0.03	0.07	102.44 (0.88)	95.21 (1.68)	100.39 (7.02)	94.20 (2.73)	103.45 (0.75)	96.65 (0.82)
artificial saliva	BFDGE·2H2O	0.03	0.07	108.59 (2.04)	110.14 (1.09)	112.13 (2.31)	80.91 (1.19)	81.19 (1.07)	85.28 (0.79)
	BADGE·2H2O	0.03	0.08	101.67 (8.76)	115.40 (1.41)	109.74 (1.94)	91.52 (8.28)	92.08 (3.79)	96.60 (1.48)
	BADGE·H2O	0.03	0.08	104.99 (2.58)	107.27 (1.21)	103.67 (7.38)	92.58 (3.29)	90.04 (8.03)	92.13 (4.72)
	BFDGE	0.03	0.07	101.17 (4.99)	103.89 (1.43)	99.27 (5.54)	91.26 (1.74)	92.16 (3.53)	100.67 (5.10)
	BADGE·H2O·HCl	0.03	0.08	101.84 (5.80)	93.80 (3.58)	92.10 (1.63)	94.52 (1.38)	93.74 (5.39)	100.67 (5.10)
	BFDGE·2HCl	0.03	0.08	105.45 (2.65)	92.83 (6.20)	94.96 (8.32)	94.28 (2.22)	106.97 (6.54)	96.76 (3.30)
	BADGE	0.02	0.07	107.68 (3.25)	98.99 (6.41)	98.00 (2.28)	92.96 (3.49)	93.40 (7.05)	100.67 (5.10)
	BADGE·HCl	0.03	0.08	112.34 (3.14)	111.21 (1.65)	111.14 (0.98)	92.92 (1.69)	95.69 (1.04)	100.67 (5.10)
	3-Ring NOGE	0.03	0.08	107.09 (1.69)	98.35 (3.19)	108.38 (3.35)	107.12 (0.45)	102.06 (0.31)	100.67 (5.10)
	BIS-DMA	0.02	0.07	97.03 (6.78)	101.85 (0.92)	116.52 (4.45)	97.74 (0.66)	105.68 (0.01)	104.29 (0.91)
5% ethanol solution	BFDGE·2H2O	0.03	0.08	100.49 (7.46)	108.48 (1.32)	91.91 (4.47)	91.42 (1.73)	82.14 (1.10)	95.36 (0.98)
	BADGE·2H2O	0.03	0.08	98.49 (4.86)	92.79 (1.86)	93.24 (0.89)	90.58 (2.01)	88.39 (4.73)	96.30 (4.07)
	BADGE·H2O	0.03	0.07	103.51 (1.24)	98.02 (1.27)	103.23 (4.72)	106.25 (2.66)	90.06 (4.26)	108.33 (8.84)
	BFDGE	0.03	0.08	100.39 (1.03)	105.24 (2.54)	98.91 (3.68)	104.47 (0.42)	98.08 (1.89)	108.75 (5.45)
	BADGE·H2O·HCl	0.03	0.08	102.92 (8.20)	92.33 (1.91)	96.90 (1.84)	94.94 (0.34)	101.54 (2.78)	96.19 (8.10)
	BFDGE·2HCl	0.03	0.07	105.81 (3.71)	99.71 (4.61)	110.73 (7.12)	94.86 (3.85)	97.50 (7.25)	102.08 (1.93)
	BADGE	0.03	0.07	105.28 (6.87)	90.50 (2.44)	91.37 (4.70)	84.15 (2.36)	82.97 (1.76)	95.08 (0.91)
	BADGE·HCl	0.03	0.08	106.93 (3.41)	93.11 (2.49)	92.62 (0.66)	95.10 (2.88)	93.21 (0.76)	94.57 (2.02)
	3-Ring NOGE	0.03	0.08	100.78 (1.29)	103.18 (7.05)	109.24 (4.71)	108.63 (2.23)	103.01 (5.51)	103.08 (3.65)
	BIS-DMA	0.03	0.08	95.48 (2.99)	93.32 (3.82)	98.34 (1.85)	97.02 (0.96)	94.18 (1.65)	96.46 (8.38)
